# Expression of miR-195 is associated with chemotherapy sensitivity of cisplatin and clinical prognosis in gastric cancer

**DOI:** 10.18632/oncotarget.21919

**Published:** 2017-10-19

**Authors:** Rui Ye, Bo Wei, Sheng Li, Wei Liu, Juntao Liu, Luan Qiu, Xuan Wu, Zhifei Zhao, Jianxiong Li

**Affiliations:** ^1^ Department of Oncology, Beidaihe Sanatorium of Beijing Military Command, Qinhuangdao 066100, Hebei, P.R. China; ^2^ Department of Radiotherapy, Chinese PLA General Hospital, Beijing 100853, P.R. China; ^3^ Department of General Surgery, Chinese PLA General Hospital, Beijing 100853, P.R. China; ^4^ Department of Biological Repositories, Zhongnan Hospital of Wuhan University, Wuhan 430071, Hubei, P.R. China; ^5^ Institute of Microbiology and Epidemiology, Academy of Military Medical Sciences, Beijing 100853, P.R. China; ^6^ Department of General Thoracic Surgery, Affiliated Hospital of Logistics University of Chinese People’s Armed Police Forces, Tianjin 300162, P.R. China; ^7^ Department of Radiotherapy, Hainan Branch of Chinese PLA General Hospital, Sanya 572000, Hainan, P.R. China

**Keywords:** miR-195, AKT3, gastric cancer, chemotherapy sensitivity

## Abstract

Gastric cancer has higher morbidity and mortality than other cancers for the low diagnosis rate and few therapies. MiR-195 has been reported to be involved in the occurrence, development and prognosis of various cancers. However, the function of miR-195 in gastric cancer remains largely unknown. Herein, the aims of this study were to probe the functional mechanism of miR-195 and its chemotherapy sensitivity as well as clinical prognosis in gastric cancer. We screened out low-expressed miR-195 through microarray analysis and further confirmed miR-195 was widely down-regulated in gastric cancer cells. Subsequently, AKT3 was identified as the direct target gene of miR-195 by target gene prediction software, dual luciferase reporter assay and western blot. Functional assays indicated that miR-195 acted as a tumor suppressor through regulating the proliferative, migrated and invasive properties of gastric cancer cells *in vitro*, and intratumoral delivery of miR-195 significantly suppressed tumor growth *in vivo*. Additionally, we also found miR-195 overexpression could enhance the chemotherapy sensitivity of cisplatin in gastric cancer cells and prolong the overall survival and progression free survival of gastric cancer patients. Collectively, our findings demonstrate miR-195 may be of great significance on early diagnosis of gastric cancer, providing the theoretical basis for prognosis and recurrence risk.

## INTRODUCTION

MicroRNAs (miRNAs) are endogenous noncoding regulatory RNAs with 17-25 nucleotides, which play vital roles in post-transcriptional gene regulation [1]. Up to now, more than 2,000 miRNAs have been registered in the “miRBase” database [2]. MiRNAs are strongly linked with biological processes consisting of cell proliferation, metastasis, differentiation, and apoptosis [3, 4]. Mounting evidence has demonstrated that multiple miRNAs are abnormally expressed in human cancers and functionally participate in regulation of various pathological processes during tumor initiation and progression [5]. Several miRNAs have been identified as either oncogenes or tumor suppressors. For example, miR-195 as a tumor-suppressive miRNA is correlated with metastases and recurrences of gastric cancer (GC) [6], breast cancer [7], and human cervical cancer [8], while miR-21 as a well-characterized oncogenic miRNA is overexpressed in breast cancer, colorectal cancer, lung cancer, GC and so on [9-12].

GC is one of the most common malignancies with a high mortality rate that ranks as the second leading cause of cancer-related deaths worldwide [13]. Available therapeutic methods are limited due to the fact that the majority of GC patients are diagnosed at advanced stages. Currently, surgery remains the main curative therapy, while perioperative and adjuvant chemotherapy with chemotherapy drugs such as fluoropyimidine (5-FU commonly used), platinum (cisplatin (DDP) commonly used), and antharcycline (doxorubicin commonly used), can improve outcome of resectable GC [14]. Over the past few years, great progresses in the diagnosis and treatment of GC have been made. However, the 5-year-survival rate in those patients is still unsatisfactory on account of its recurrence and metastasis [15, 16]. Just as mentioned above, miR-195 as a tumor-suppressive miRNA is associated with metastasis and recurrence of GC [8]. As the role of miR-195 in tumorigenesis is gradually discovered, the great significance of this molecule associated with clinical diagnosis and prognosis has been attracting more and more attentions from scholars.

The serine/threonine kinase AKT, a major downstream mediator of the phosphoinositide 3-kinase (PI3K)-pathway, is known to be related with disease free survival and tumor progression [17, 18]. AKT3 is one of the three highly homologous AKT isoforms, which contributes to the formation of metastases. Nowadays, emerging data have indicated that AKT3 is also strongly linked with several other malignancies, including GC, ovarian cancer, glioblastoma, breast cancer, triple negative breast cancer, and hepatocellular carcinoma [19-23].

In the present study, the functional role of miR-195 in GC progression was firstly evaluated *in vitro* and *in vivo*, and then its relationship with chemotherapy sensitivity and clinical prognosis was analyzed.

## RESULTS

### MiR-195 was remarkably down-regulated in GC samples

**Figure 1:**
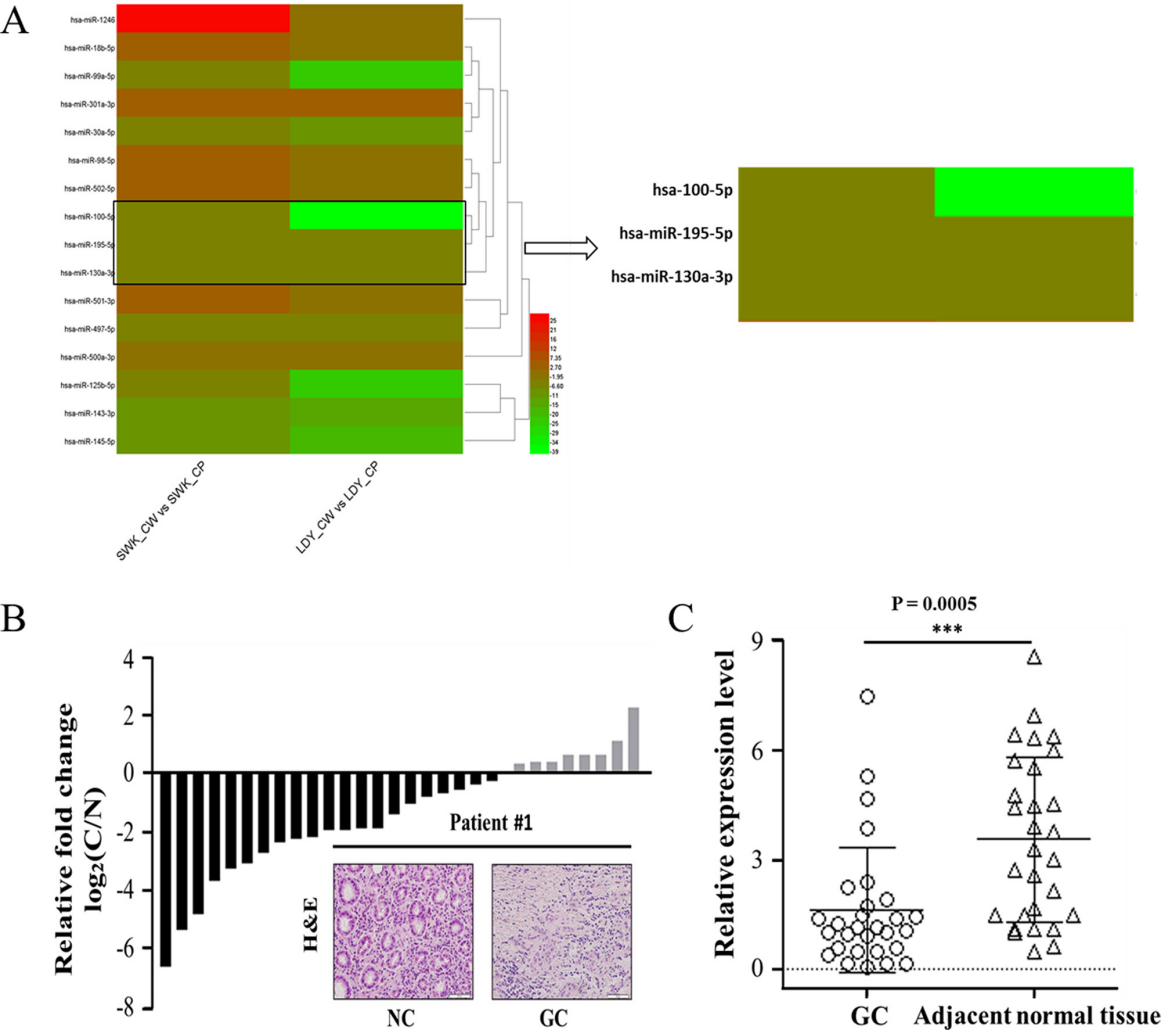
MiR-195 was remarkably down-regulated in GC samples **(A)** Heat maps of differentially expressed miRNAs between GC tissues and adjacent normal tissues. LDY_CP, LDY_CW, SWK_CP and SWK_CW are all sample IDs, wherein CP represents adjacent normal tissues and CW refers to GC tissues. Red represents higher expression and green represents lower expression. **(B)** RT-qPCR analysis of miR-195 expression in twenty-nine pairs of GC tissues and H&E stain of GC and adjacent tissues of patient#1. **(C)** The statistical analysis of miR-195 relative expression level in tumor tissues and adjacent tissues. Horizontal line represents the average value for each group. NC, negative control; GC, gastric cancer; RT-qPCR, real-time quantitative polymerase chain reaction; ***, *P <* 0.001.

Two tumor tissues and adjacent tissues were used to fulfill microarray analysis and fold change method was employed to conduct differential analysis of miRNA expression levels in the two group tissues. Clear-cut distinction was drawn in the sixteen miRNA expression levels (Figure 1A, fold change > 2). MiR-195 attained our focus from these significantly differentially expressed miRNAs for the fact that miR-195 expression was evidently down-regulated and its relevant research in GC has largely unreported. According to microarray analysis and miRBase, its mature sequence was confirmed, which was described in corresponding Materials and methods part. Subsequently, real-time quantitative polymerase chain reaction (RT-qPCR) was performed on twenty-nine tumor tissues and adjacent tissues to detect the mRNA expression of miR-195. Of those twenty-nine tumor tissues, miR-195 expression was down-regulated in twenty-one tissues (Figure 1B), accounting for 72.4%. In addition, the statistical analysis revealed an evident statistical significance in the relative expression level of miR-195 between tumor tissues and adjacent tissues (Figure 1C, *P* < 0.001).

### AKT3 was a direct target of miR-195

**Figure 2:**
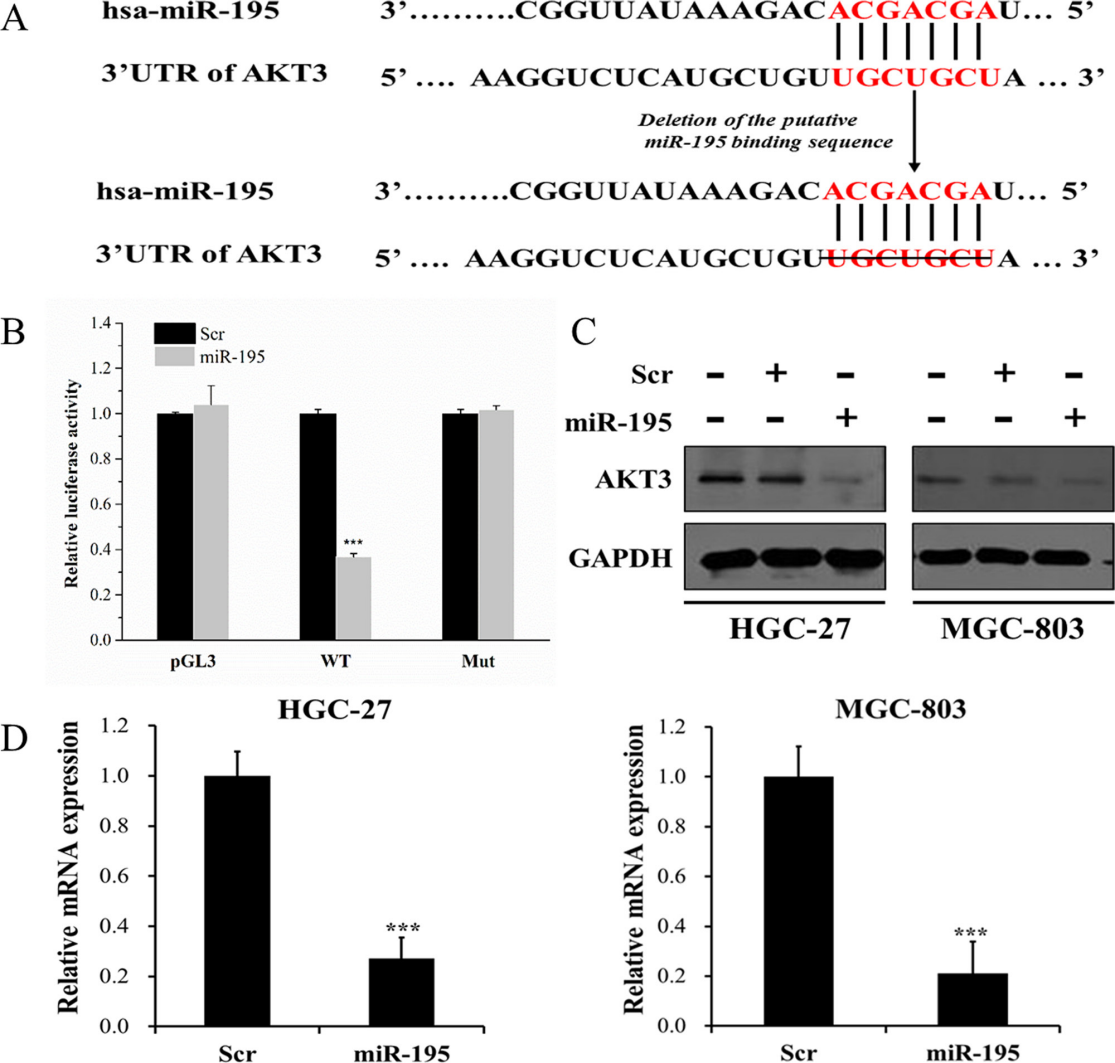
AKT3 was identified as a direct target of miR-195 **(A)** Schematic representation of AKT3 3’-UTR showing putative miR-195 target sites. **(B)** The results suggested that the relative luciferase activity was evidently suppressed by miR-195 overexpression in cells transfected with pMIR-AKT3-WT, while it showed no difference of luciferase activity in cells transfected with pMIR-AKT3-Mut. **(C)** Western blot showed that the AKT3 protein expression was sufficiently decreased in miR-195 mimic group when compared with Scr group, whereas it showed no difference between Scr group and untreated group at AKT3 protein expression level. **(D)** The RT-qPCR assay indicated that the AKT3 mRNA expression in miR-195-treated group was significantly lower than that in Scr group. Error bars represented standard deviation obtained from three independent experiments and all the data were shown as mean ± SD. WT, wild type; Mut, mutation; Scr, scramble; RT-qPCR, real-time quantitative polymerase chain reaction; ***, *P <* 0.001.

Since the fact that miRNAs exert their molecular functions by mediating the target genes thereof, we explored the candidate target genes of miR-195 using Targetscan, MIRanda and PicTar. Ten putative target genes, consisting of CD28, BCL2L2, MAP2K1, KLC2, NRAS, HOXA10, HOXA3, BCL9L, STX1A, AKT3, were primarily selected according to dual luciferase reporter assays (Supplementary Figure 1). In concrete, taking AKT3 for instance, pMIR-AKT3-WT and pMIR-AKT3-Mut (Figure 2A) were constructed. Afterwards, the pRL-TK vector with pGL3 vector, pMIR-AKT3-WT vector or pMIR-AKT3-Mut vector were in the combination of miR-195 mimic or scramble (Scr) to co-transfect 293T cells. The results suggested that the relative luciferase activity was evidently suppressed by miR-195 overexpression in cells transfected with pMIR-AKT3-WT (*P <* 0.001), while it showed no difference of luciferase activity in cells transfected with pMIR-AKT3-Mut (Figure 2B). So were the cases in BCL2L2 (Supplementary Figure 1, *P* < 0.01) and NRAS (Supplementary Figure 1, *P* < 0.05). These results covered that AKT3, BCL2L2 and NRAS were likely to be direct targets of miR-195 in GC. Subsequently, primary RT-qPCR assay revealed that AKT3 mRNA activity was significantly suppressed in miR-195 mimic group (Supplementary Figure 2, *P* < 0.001), while no clear-cut distinction was drawn among BCL2L2 group and NRAS group (Supplementary Figure 2). It was therefore AKT3 carried the first possibility to be the direct target of miR-195. To further verify this finding, miR-195 mimic or Scr was transfected into GC cell lines HGC-27 and MGC-803, respectively. Western blot showed that the AKT3 protein expression was sufficiently decreased in miR-195 mimic group when compared with Scr group (*P <* 0.001), whereas it showed no difference between Scr group and untreated group at AKT3 protein expression level (Figure 2C). Similar to primary RT-qPCR assay, the obtained results indicated that the AKT3 mRNA expression in miR-195 mimic group was significantly lower than that in Scr group (Figure 2D, *P <* 0.001), certifying that AKT3 was a direct target of miR-195 in GC.

### MiR-195 inhibited cell proliferation, migration and invasion *in vitro* GC cells

**Figure 3:**
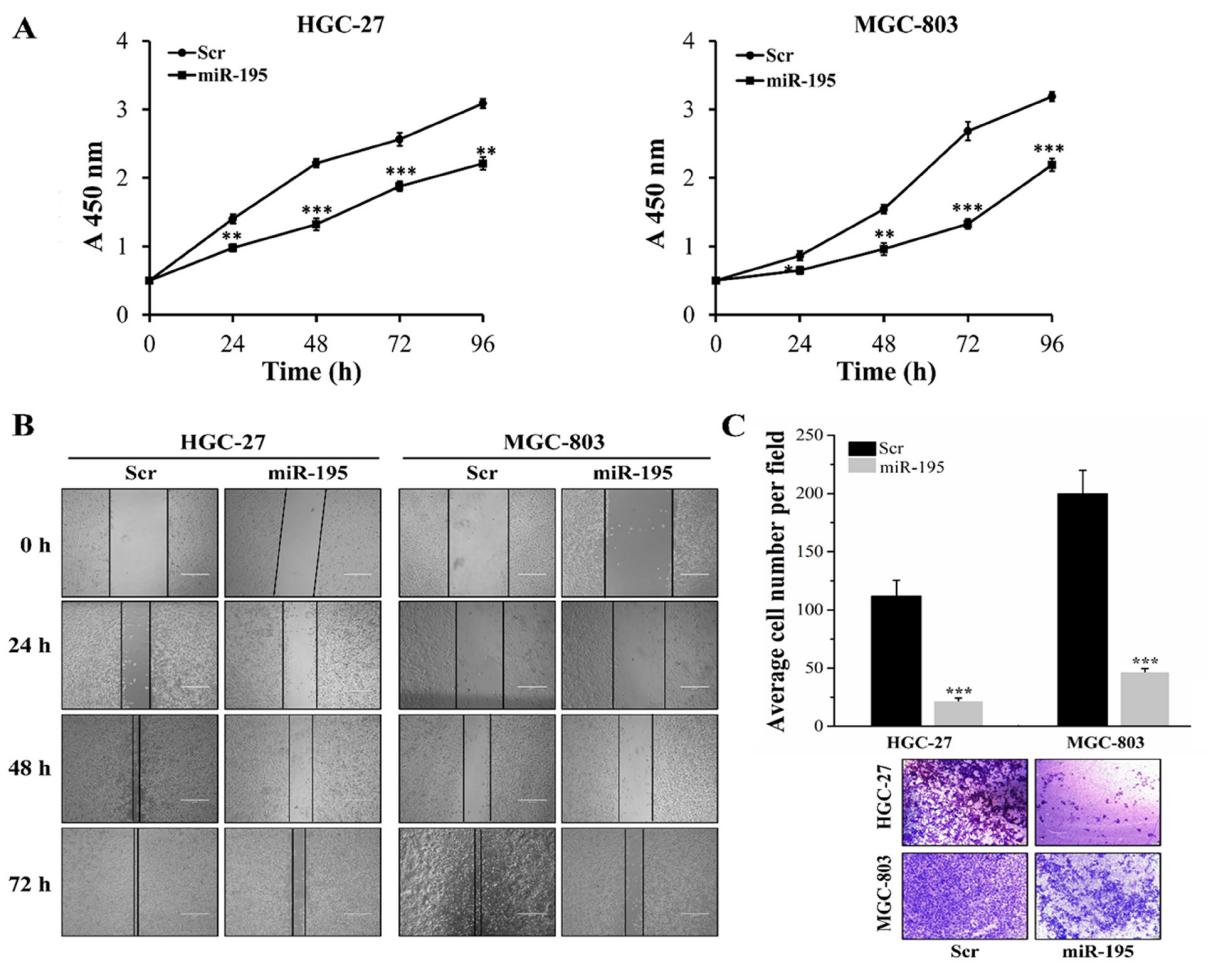
MiR-195 inhibited cell proliferation, migration and invasion *in vitro* GC cells **(A)** CCK-8 assays revealed that the proliferative abilities of HGC-27 and MGC-803 cells transfected with miR-195 mimic were significantly suppressed compared with those transfected with the Scr. Scratch wound-healing assays **(B)** and transwell assays **(C)** revealed that both the migrated and invasive properties of cells transfected with miR-195 mimic were remarkably impaired when compared with those transfected with the Scr. Error bars represented standard deviation obtained from three independent experiments and all the data were shown as mean ± SD. All the scale bars are 500 μm. GC, gastric cancer; CCK-8, Cell Counting Kit-8; Scr, scramble; *, *P <* 0.05; **, *P <* 0.01; ***, *P <* 0.001.

To investigate the functional roles of miR-195 in GC, cell proliferation, migration, and invasion assays were conducted in HGC-27 and MGC-803 cells transfected with miR-195 mimic or Scr. Cell Counting Kit-8 (CCK-8) assays revealed that the proliferative abilities of HGC-27 and MGC-803 cells transfected with miR-195 mimic were significantly suppressed compared with those transfected with the Scr (Figure 3A, *P <* 0.05). Similarly, we found that both the migrated and invasive properties of cells transfected with miR-195 mimic were remarkably impaired when compared with those transfected with the Scr via scratch wound-healing assays and transwell assays (Figure 3B, 3C, *P <* 0.001).

### MiR-195 showed a visible inhibitory impact on tumor growth *in vivo*

**Figure 4:**
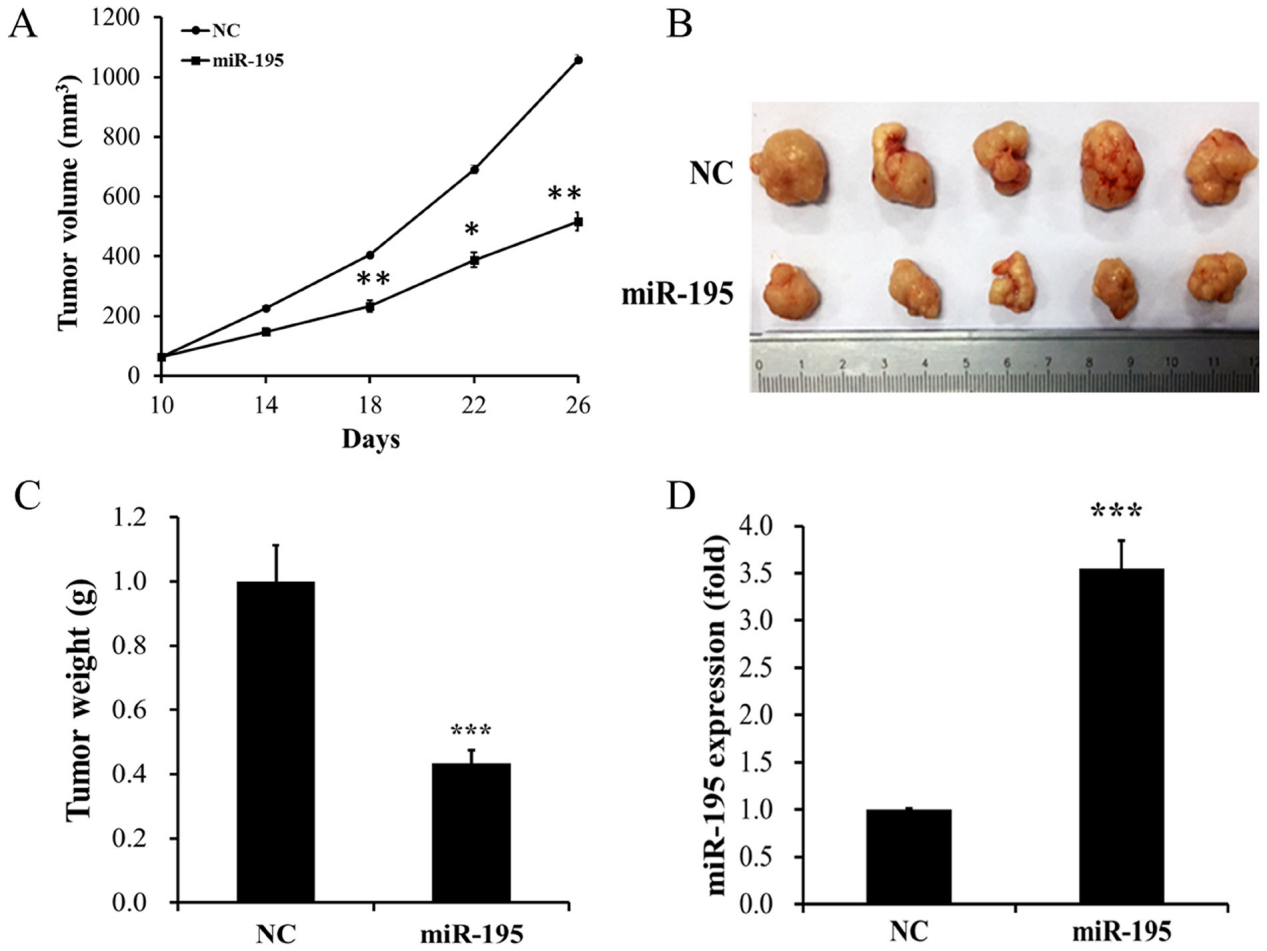
MiR-195 showed a visible inhibitory impact on tumor growth *in vivo* **(A)** Growth curve of tumor volumes presented a slower growth in miR-195-treated group than those in NC group. The first intratumor injection of miR-195 was performed once palpable tumors (average volume of 62.5 mm^3^) were detected (usually on the 10th day). **(B)** Representative photograph of xenograft tumors showed that tumor size in miR-195-treated group was significant larger than that in NC group. **(C)** Tumor weight increased evidently in NC group when compared with that in miR-195-treated group. **(D)** RT-qPCR of xenograft tumors revealed that miR-195 expression was up-regulated in miR-195-treated group. NC, negative control; RT-qPCR, real-time quantitative polymerase chain reaction; *, P < 0.05; **, P < 0.01; ***, P < 0.001.

In this section, the impact of miR-195 overexpression on GC progression *in vivo* was examined using intratumoral delivery approach. MGC-803 cells were injected subcutaneously into nude mice, and then miR-195 agomir or negative control (NC) was injected on the 10^th^ day. Tumor volume was calculated every four days. The results indicated that tumor volumes presented a slower growth in miR-195-treated group than in NC group (Figure 4A, *P <* 0.05). The mice were killed due to the presence of large tumor in NC group on the 26^th^ day. Representative photograph of xenograft tumors showed that tumor sizes in miR-195-treated group were significant smaller than those in NC group (Figure 4B). Meanwhile, we observed that tumor weight increased evidently in NC group when compared with that in miR-195-treated group (Figure 4C, *P <* 0.05). In addition, RT-qPCR of xenograft tumors revealed that miR-195 expression was up-regulated in miR-195-treated group (Figure 4D, *P <* 0.05). Taken together, these findings verified that miR-195 overexpression significantly suppressed tumor growth *in vivo*.

### Overexpression of miR-195 enhanced the chemotherapy sensitivity of DDP in GC cells

**Figure 5:**
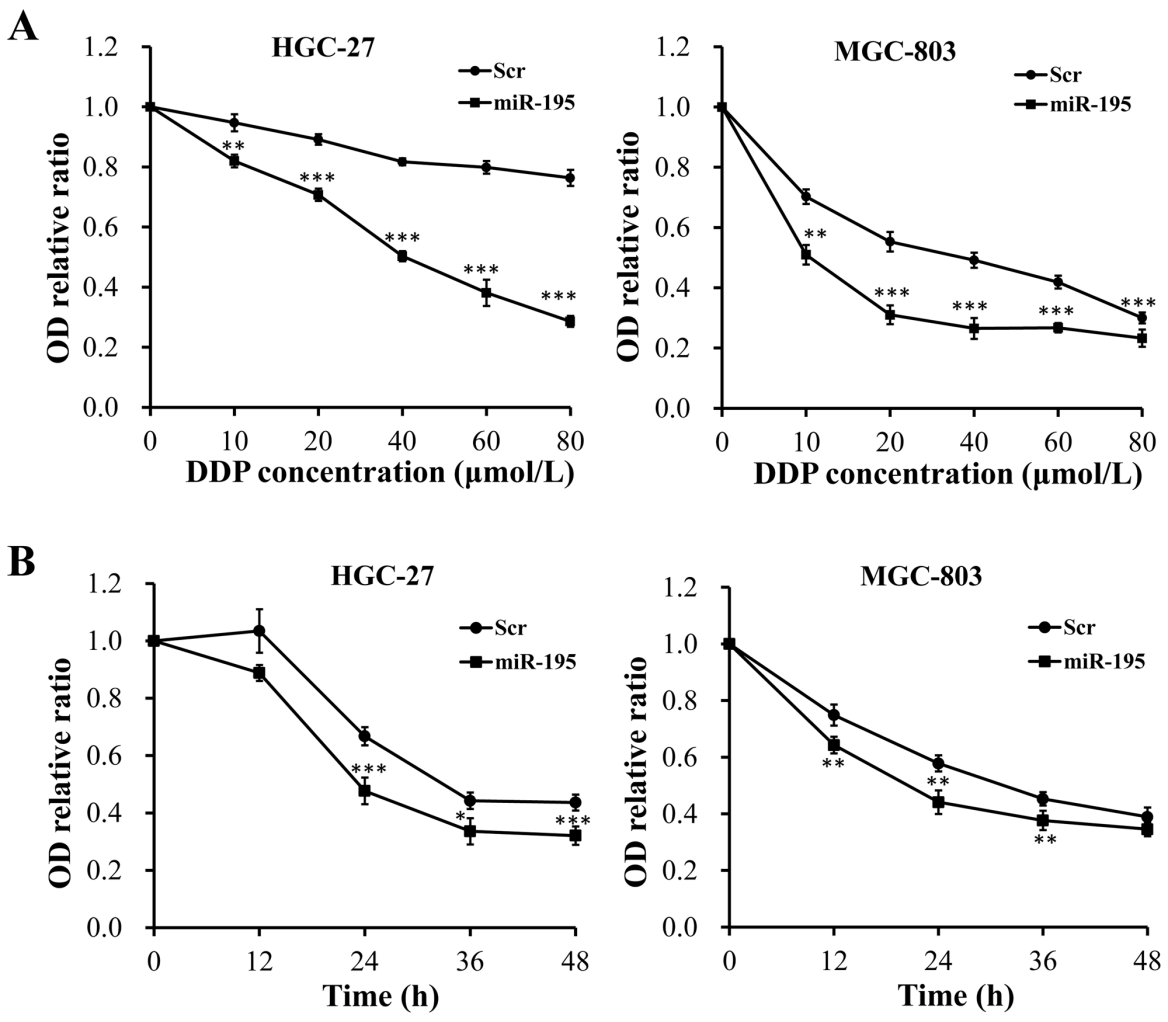
Overexpression of miR-195 enhanced the chemotherapy sensitivity of DDP in GC cells **(A)** CCK-8 assays indicated that overexpression of miR-195 remarkably impaired the GC cell viability at each concentration of DDP when compared with the Scr. **(B)** CCK-8 assays showed that miR-195 could enhance the chemotherapy sensitivity of DDP at varied times in GC cells. Error bars represented standard deviation obtained from three independent experiments and all the data were shown as mean ± SD. DDP, cisplatin; GC, gastric cancer; CCK-8, Cell Counting Kit-8; Scr, scramble; *, *P <* 0.05; **, *P <* 0.01; ***, *P <* 0.001.

To discover the influence of DDP concentration on the chemotherapy sensitivity of miR-195 in GC cells, we transfected miR-195 mimic or Scr into HGC-27 and MGC-803 cells. Afterwards, different concentrations of DDP (i.e., 0, 10, 20, 40, 60 and 80 μmol/L) were added into each group. Then, the viability of those two cell lines treated with different concentrations of DDP was evaluated using CCK-8 assays. As depicted in Figure 5A, overexpression of miR-195 remarkably impaired the GC cell viability at each concentration of DDP when compared with the Scr (Figure 5A, *P <* 0.01), indicating that miR-195 could enhance the chemotherapy sensitivity of DDP at different concentration in GC cells. In addition, we also noticed that the IC50 value of DDP was 50 μmol/L.

To further determine the influence of functional time on the chemotherapy sensitivity of miR-195 for DDP in GC cells, CCK-8 assays were respectively conducted after adding 50 μmol/L (IC50) DDP for varied times (i.e., 0 h, 12 h, 24 h, 36 h and 48 h). Similarly, we found that miR-195 could enhance the chemotherapy sensitivity of DDP at varied times in GC cells (Figure 5B, *P <* 0.05).

### The correlation analyses between miR-195 expression and clinical manifestation and prognosis of patients with GC

**Figure 6:**
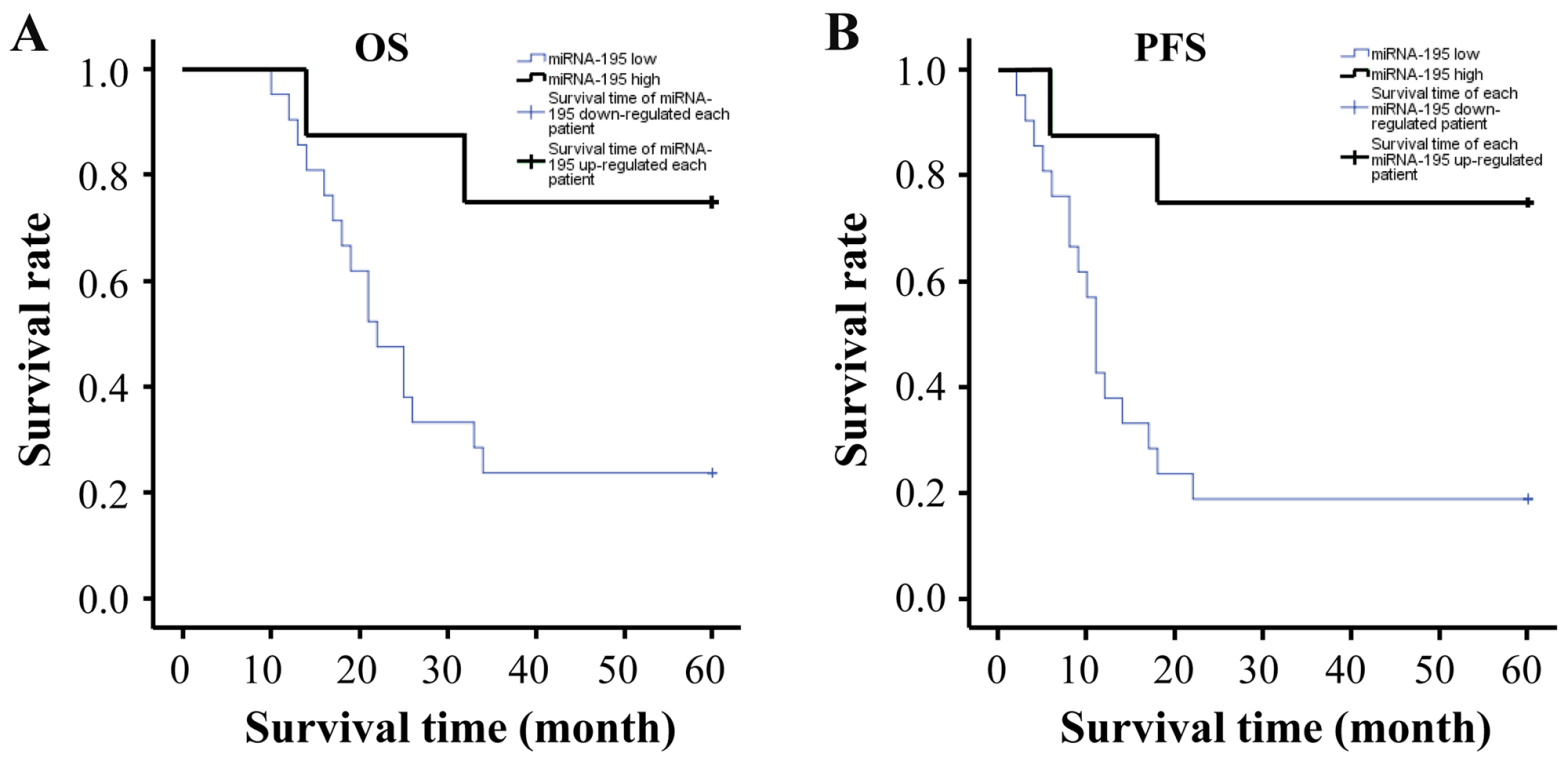
The correlation analyses between miR-195 expression and prognosis of patients with GC The results indicated that **(A)** the MST of OS for miR-195 high-expression patients was over sixty months and for low-expression was about twenty-two months (*P =* 0.023), while **(B)** the corresponding MST of PFS was more than sixty months and around eleven months, respectively (*P =* 0.010). MST, median survival time; OS, overall survival; PFS, progression free survival.

To examine the correlation between miR-195 and clinicopathological characteristics of patients with GC, the clinical data of twenty-nine patients were collected (Table 1), including gender, age, vascular invasion, tumor location, Borrmann type, tumor node metastasis (TNM) stage and grade. No clear-cut correlation was observed between miR-195 expression and above clinicopathological features. So was the case in the AKT3 expression (Supplementary Table 1). Furthermore, we performed the correlation of miR-195 expression with overall survival (OS), and progression free survival (PFS). The results indicated that the median survival time (MST) of OS for miR-195 high-expression patients was over sixty months and for low-expression was about twenty-two months (Figure 6A, *P =* 0.023), while the corresponding MST of PFS was respectively more than sixty months and around eleven months (Figure 6B, *P =* 0.010). Taken together, it could be concluded that miR-195 expression was related with both OS and PFS.

**Table 1:** Correlation analysis between miR-195 expression in GC and clinicopathological characteristic

Clinicopathological characteristics		Number of cases	Mean^1^	SD^2^	P-value
Gender	Male	22	-1.718	2.202	0.181
	Female	7	-0.769	1.305	
Age	≤60	16	-1.567	2.264	0.820
	>60	13	-1.393	1.820	
Vascular invasion	Positive	15	-0.971	1.736	0.166
	Negative	14	-2.044	2.259	
Tumor location	Cardia	5	-3.011	2.202	
	Gastric body	3	0.137	1.889	
	Gastric antrum	21	-1.359	1.877	
Borrmann type	I+II	15	-1.559	1.693	0.854
	III+IV	14	-1.414	2.427	
pT stage	T1+T2	1	-2.374		
	T3+T4	28	-1.457		
pN stage	N0+N1	12	-1.245	1.743	0.581
	N2+N3	17	-1.662	2.267	
pM stage	M0	28	-1.563		
	M1	1	0.591		
pTNM stage	I+II	6	-1.204	2.079	0.716
	III+IV	23	-1.563	2.074	
Histologic grade	well	10	-2.500	2.577	0.106
	poor	19	-0.957	1.519	

^1^ Mean of Log_2_ (C/N); ^2^ Standard Definition of Log_2_ (C/N); C: normalized expression of cancer tissues; N: normalized expression of adjacent noncancerous tissues.

## DISCUSSION

Recently, numerous evidences have demonstrated that miRNAs expression has significant differences in tumor tissues and normal tissues. Those differentially expressed miRNAs are frequently located at fragile sites which are generally implicated in cancer occurrence [5]. Meanwhile, plentiful studies have proved that that miRNAs exist in the variable genome involved in cancers, which makes researchers focus on the role of miRNAs in the process of cancer development [24]. For example, the expression profile of miR-21 was identified by miRNA microarray analysis and its expression was obviously up-regulated in lung cancer tissues, exhibiting a high association with tumorigenesis and various prognosis factors [11, 25]. Another example is that twenty nine miRNAs were identified to be expressed differentially in breast cancer cells, and more importantly, those ectopic expression miRNAs could up-regulate or down-regulate their target genes to down-regulate the anti-apoptotic gene expression, contributing to the apoptosis of breast cancer cells [26, 27].

Previous studies have deeply investigated the regulation mechanisms of miR-195 in a variety of common cancers, such as hepatocellular carcinoma [28], colorectal cancer [29], non-small cell lung cancer [30] and breast cancer [6, 31]. However, the research about the regulation mechanisms of miR-195 in GC is just emerging. It has been verified that miR-195 expression was down-regulated in GC tissue when compared with its corresponding paracancerous tissue [32]. Meanwhile, some studies have shown the methylation modulation may be one of the mechanisms inducing the decreased expression of miR-195, who may play a role in tumor inhibition by regulating CDK6 kinase and vascular endothelial growth factor (VEGF) [8]. Despite of abundant investigations on the relationship between miR-195 expression and GC, the specific regulatory mechanism of miR-195 in GC has not been further explored, and the target gene of miR-195 has not been clearly validated. Therefore, this study was performed to uncover the specific regulatory mechanism of miR-195 in GC.

In present study, we first screened out low-expressed miR-195 based on the microarray analysis and confirmed that miR-195 was widely down-regulated in GC cells by RT-qPCR assays, which were consistent with previous studies [8, 32]. Additionally, a series of functional assays indicated that miR-195 was regarded as a tumor suppressor through regulating the proliferative, migrated and invasive properties of GC cells *in vitro*. The *in vivo* intratumoral delivery of miR-195 into subcutaneous xenografts significantly alleviated tumor growth. The miR-195 expression was obviously higher in miR-195-treated tumors (fold change > 2) than in NC tumors, highlighting the role of miR-195 in tumor progression *in vivo*. Generally, the biological functions of miRNAs were determined by their downstream target genes. Hence, three prediction softwares (Targetscan, MIRanda and PicTar) were used to screen the direct target gene of miR-195. Ten putative target genes, consisting of CD28, BCL2L2, MAP2K1, KLC2, NRAS, HOXA10, HOXA3, BCL9L, STX1A, AKT3, were primarily selected according to dual luciferase reporter assays and RT-qPCR assay. Ultimately, AKT3 was verified to be the direct target of miR-195 via dual luciferase reporter assays, Western blot coupled with RT-qPCR assay. It is well-known that AKT3 is an important member of PI3K/AKT signaling pathway [33].

In recent studies, it has been suggested that PI3K/AKT signaling pathway plays a significant role in the occurrence and development of multiple cancers, including lung cancer, GC, as well as head and neck cancer [34]. However, the correlation between AKT3 and miRNAs has rarely been reported. In the present paper, we affirmed that AKT3 was a direct target gene of miR-195 for the first time and speculated that miR-195 was likely to inhibit the proliferative, migrated and invasive properties of GC cells through PI3K/AKT signaling pathway.

Recently, emerging evidences have shown that miR-195 can enhance the chemotherapy sensitivity in various cancers. For example, it has been reported that miR-195 plays a positive effect on the chemotherapy sensitivity of doxorubicin in colon cancer cells by targeting the first binding site of BCL2L2 mRNA [35]. Another study about that miR-195 increases the chemotherapy sensitivity of 5-FU in human hepatocellular carcinoma cells by targeting BCL-w has also been reported [36]. However, the study concerning the effect of miR-195 on chemotherapy sensitivity in GC has not been reported up to now. Yan B et al. has confirmed that miRNA-29b can increase the chemotherapy sensitivity of DDP in prostate cancer cells by targeting AKT3 [37]. In this study, we confirmed that AKT3 was the direct target gene for miR-195 in GC cells. We were therefore tempting to speculate that miR-195 may be linked to chemotherapy sensitivity of DDP. Accordingly, related experiments were performed and the obtained results revealed that miR-195 could enhance the chemotherapy sensitivity of DDP in GC cell lines at different times. We conclude that miR-195 has a promoting effect on chemotherapy sensitivity in GC cells, which provides a theoretical and experimental basis for the exploration of miR-195 new targets. Additionally, the relationship between miR-195 and the OS and PSF of postoperative patients with GC also has not been illustrated. Hereby, we performed the correlation analysis between miR-195 expression and clinical data. The results showed that miR-195 expression was remarkably associated with the OS and PSF of postoperative patients with GC and the OS of patients with high-expressed miR-195 was evidently prolonged when compared with low-expressed group.

In conclusion, these data are consistent with the possibility that miR-195 plays a critical functional role in suppressing tumor progression in human GC *in vitro* and *in vivo*, probably exerting its role by modulating its target gene AKT3. Meanwhile, miR-195 has a significant effect on the chemotherapy sensitivity and clinical prognosis. Taken together, our findings indicate that miR-195 is promising to act as a therapeutic target for treatment and a biomarker for progression of human GC.

## MATERIALS AND METHODS

### Patient samples

Specimens of both GC tissues and corresponding adjacent normal tissues (>5 cm away from cancer tissues) were collected from 29 patients who received initial surgery in Chinese PLA General Hospital from 2010 to 2011. The utilizations of the tumor samples for research were approved by the ethical committee of Chinese PLA General Hospital prior to collection. In all patients, everyone had provided informed consent and no one had undergone therapy before resection. All collected samples were frozen immediately in liquid nitrogen after resection or kept in a refrigerator at -80°C.

### Cell culture

The human GC cell lines HCG-27, MGC-803 and 293T were all purchased from the Cell Centre of Peking Union Medical College Hospital. Cells were cultured in Dulbecco’s modified Eagle’s medium (DMEM) (HyClone, USA) supplemented with 10% fetal bovine serum (Hangzhou, China), 100 IU/ml penicillin and 100 μg/ml streptomycin (Gibco, Invitrogen, Shanghai, China) in a humid atmosphere containing 5% CO_2_ at 37°C.

### Plasmid construction and transfection

Cells were plated into 24-well culture plate at a density of 5×10^4^ cells pre well and cultured to attain a concentration of 70%∼80% prior to transfection. Afterwards, transfection was performed with Lipofectamine 2000 (Invitrogen, Carlsbad, CA, USA) following the manufacturer’s instruction. The sequences used in this part were presented as follows: hsa-miR-195 mimic, 5’-UAGCAGCACAGAAAUAUUGGC-3’ (sense); NC miRNA mimic, 5’-UUCUCCGAACGUGUCACGUTT-3’ (sense). At 24 h after transfection, the cells were harvested for further functional assays.

### RNA extraction and real-time quantitative polymerase chain reaction (RT-qPCR)

According to the manufacturer’s protocol, total RNA was isolated from GC cell lines using Trizol reagent (Invitrogen, Carlsbad, CA, USA). To detect mRNA, first-strand cDNA was synthesized by PrimeScript RT reagent kit (TaKaRa, Dalian, China). RT-qPCR was then performed using the ABI PRISM 7500 Fast Real–Time PCR System (Perkin Elmer/Applied Biosystems, Rotkreuz, Switzerland) with SYBR Premix Ex Taq™ II (Takara) according to the manufacturer’s instructions. U6 snRNA was used as an internal control to normalize the experimental results. As for miRNA detection, cDNA is reverse transcribed from total RNA samples using small RNA-specific, stem-loop RT primer from the TaqMan small RNA Assays and reagents from the TaqMan microRNA reverse transcription kit. MiR-195 levels were then detected using a TaqMan microRNA kit (Applied Biosystems) and normalized to U6 snRNA. The relative amount of miRNA was calculated using △△Ct. The Assay ID of miR-195 probe and U6 snRNA probe provided by ABI (USA) were 000494 and 001973, respectively. The sequences used in this work were listed as follows: AKT3 forward primer, 5’-TGAAGTGGCACACACTCTAACT-3’; AKT3 reverse primer, 5’-CCGCTCTCTCGACAAATGGA-3’; U6 RT primer, 5’-AAAATATGGAACGCTTCACGAATTTGG-3’; U6 forward primer, 5’-CTCGCTTCGGCAGCACATATACT-3’; U6 reverse primer, 5’-ACGCTTCACGAATTTGCGTGTC-3’. Each reaction was performed three times, independently.

### Luciferase reporter assay

Luciferase reporter assay was performed in 293T cells to confirm whether AKT3 was a direct target of miR-195. The 3’-untranslated regions (3’-UTR) of AKT3 containing the miR-195 binding site was cloned into the downstream of luciferase gene to generate pMIR-AKT3-WT vector, and the 3’-UTR without miR-195 binding site was used to construct pMIR-AKT3-Mut vector. Afterwards, the pRL-TK vector, pMIR-AKT3-WT vector or pMIR-AKT3-Mut vector were in the combination of miR-195 mimic or Scr to co-transfect 293T cells. The pGL3 vector group was served as control. Transfected cells were seeded into 24-well culture plate. After 48 h of transfection, luciferase activity was detected using the Dual Luciferase Reporter Assay System (Qiagen, Germany).

### Western blot

Cells were collected after 24 h of transfection, and total cells were washed thrice with phosphate-buﬀered saline (1×PBS). The addition of 100 μl lysis buffer in each well was to obtain total cell lysates and the process of lysis was performed on ice for 30 min. The sample was then centrifuged for 10 min at 12000 rpm. The supernatant was removed and its protein concentration was measured according to the BCA kit instructions. Total cellular proteins were isolated and loaded using 10% SDS-PAGE. The primary antibodies (AKT3, 1:1000, Abcam, England; GAPDH, 1:1000, Abcam, England) were incubated at 4°C overnight. Afterwards, AKT3 was incubated with the goat anti-rabbit IgG (1:10000, Abcam, England) labeled with horseradish peroxidase, and GAPDH was with goat anti-mouse IgG (1:10000, Abcam, England) labeled with horseradish peroxidase for one hour at room temperature. Immunoreactivity was measured by an ECL kit (Amersham, Sweden).

### Cell migration assays

Scratch wound-healing assays were carried out to examine GC cell migration. After 24 h of post-transfection, cells were plated into 12-well culture plate to achieve a confluent monolayer. Then, uniform wounds were scraped with a sterile 200 μl pipette tip in 12-well culture plate, and each well was washed thrice with 1×PBS to remove floating cells. The initial distance (0 h) and the residual distance (24, 48 and 72 h after scratch) were inspected microscopically.

### Cell proliferation assays

CCK-8 was conducted to detect the cell proliferative properties of transfected cells according to the manufacturer’s recommendations (Dojindo Inc., Kumamoto, Japan). Transfected cells were seeded into 96-well culture plates with 100 μl culture medium. At a regular interval (0, 12, 24, 36 and 48 h) of treatment with cisplatin (DDP), each well was added 10 μl CCK-8 solution, respectively. After 2 h of further incubation with CCK-8 solution, the visual density of each well was measured at 450 nm wavelength by MK3 Microplate Reader (Bio-Rad, USA), and the relative number of optical cells in each well was counted.

### Cell invasion assays

Transwell assays were used to evaluate invasive abilities of GC cells *in vitro*. Cells were transferred into the upper wells of 24-well transwell matrigel chambers (BD Biosciences, USA) after 48 h of transfection. DMEM medium supplemented with 10% fetal bovine serum served as chemoattractant filling in the lower wells. After 48 h of incubation, non-invading cells on the upper surface of membranes were removed, and cells that had invaded to the lower surface of membranes were stained with Giemsa for 30 min and calculated under a light microscope in ten random fields.

### *In vivo* intratumoral delivery of miR-195

MGC-803 cells (1×10^6^) were resuspended in 100μL of PBS and implanted into the nude mice. Once palpable tumors (average volume of 62.5 mm^3^) were detected (usually on the 10^th^ day), 2 nmol of miR-195 agomir or NC was diluted into 25 μl of autoclaving sterilized PBS, and then the mixture solution was intratumorally injected into nude mice (*n* = 5) through muti-point injection at 3-day interval. A total of four interval injections were conducted. Tumors were measured using a digital caliper. The equation of calculating tumor volumes calculated was as follows: *V (mm*^*3*^*) = L×P*^*2*^*/2*, where *L* was the largest diameter and *P* was the perpendicular diameter. In 26 days after administration, mice were euthanized and tumor tissues were collected for continuous experiments. All animal care was in accordance with institutional guidelines.

### MiRNA microarray analysis

The tumor tissues and adjacent normal tissues of two GC patients were collected for the microarray analysis. Microarray analyses were performed by Bioassay Laboratory of CapitalBio Corporation (Beijing, China). RNA extraction, the purification and dephosphorylation intersected with labeling reaction of total RNA were all performed according to the manufacturer’s instructions. In the whole process, labeling spike-in RNA and Hyb spike-in RNA (Agilent Technologies, Inc., Santa Clara, CA, USA) were added and used as quality control. Microarray slides were scanned and microarray images were automatically analyzed using Feature extraction™ software, version 10.7 (Agilent Technology, Inc., Santa Clara, CA, USA). GeneSpring (Agilent Technologies, Inc., Santa Clara, CA, USA) was used to normalize the data and perform variation analysis. Differentially expressed miRNAs were screened out according to fold change (FC) > 2 and the related heat maps were drawn based on the tools reported by Deng W, et al [38].

### Statistical analysis

Each experiment was repeated three times, and all values were presented as the means ± standard deviation (SD). Unless otherwise indicated, T-test and ANOVA was employed respectively to analyze the difference in two groups and multiple groups (>2). Chi-square test was used for analyzing the classification variables between different groups. Kaplan–Meier and log-rank test were used to perform survival analysis. SPSS version 18.0 (SPSS, Chicago, IL) was applied to complete statistical analysis. Value of *P <* 0.05 was considered statistically significant for all statistical analyses.

## SUPPLEMENTARY MATERIALS FIGURES AND TABLE



## Abbreviations

(miRNAs): microRNAs
(GC): gastric cancer
(PI3K): phosphoinositide 3-kinase
(RT-qPCR): real-time quantitative polymerase chain reaction
(Scr): scramble
(NC): negative control
(CCK-8): Cell Counting Kit-8
(DDP): Cisplatin
(TNM): tumor node metastasis
(OS): overall survival
(PFS): progression free survival
(MST): median survival time
(VEGF): vascular endothelial growth factor
(PBS): phosphate-buﬀered saline
(DMEM): Dulbecco’s Modified Eagle’s Medium
(SD): standard deviation
