# Supplementary material for: Expression of miR-195 is associated with chemotherapy sensitivity of cisplatin and clinical prognosis in gastric cancer
Source: Oncotarget. 2017 Oct 19;8(57):97260–72. doi: 10.18632/oncotarget.21919 (PMC5722560; doi:10.18632/oncotarget.21919)
Supplement: Supplementary file 1 [file oncotarget-08-97260-s001.pdf]

Expression of miR-195 is associated with chemotherapy sensitivity of cisplatin and clinical prognosis in gastric cancer

SUPPLEMENTARY MATERIALS

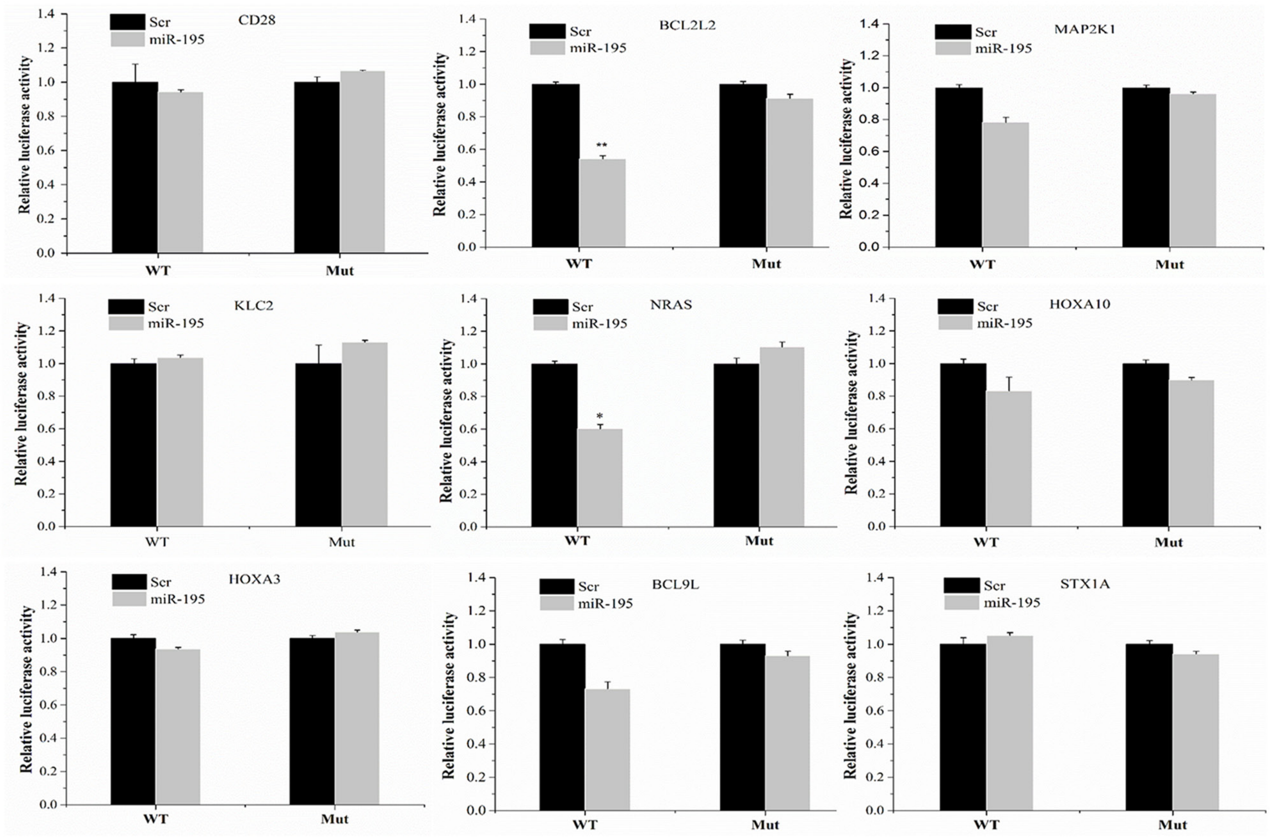

Supplementary Figure 1: Dual luciferase reporter assays of the remaining nine putative target genes. Error bars represented standard deviation obtained from three independent experiments and all the data were shown as mean ± SD. WT, wild type; Mut, mutation; Scr, scramble; *, $P < 0.05$; **, $P < 0.01$

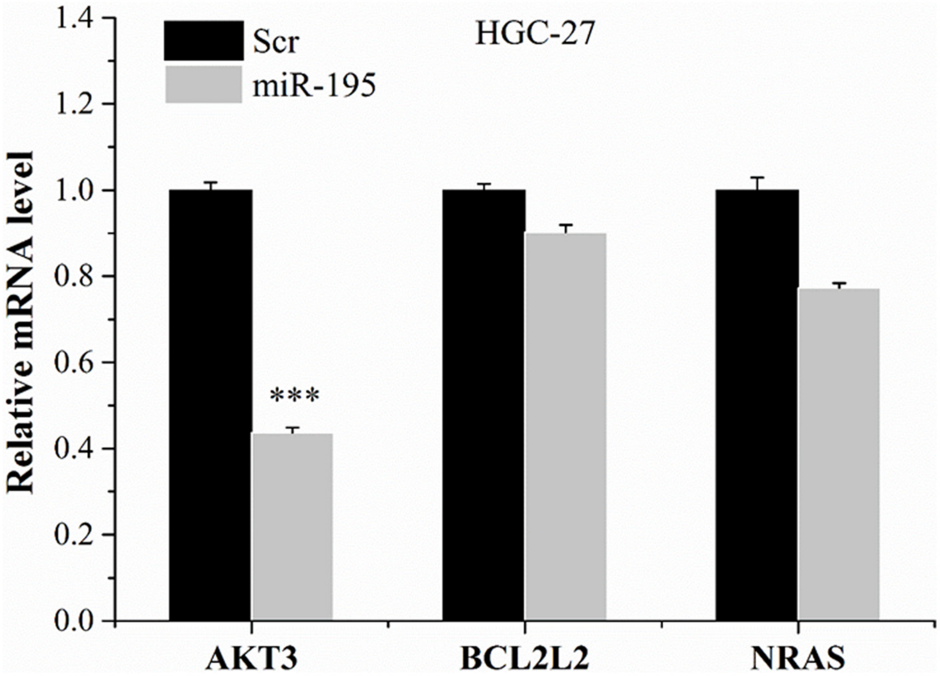

Supplementary Figure 2: The relative mRNA activity of selected three putative target genes in GC cell line. Error bars represented standard deviation obtained from three independent experiments and all the data were shown as mean ± SD. Scr, scramble; ***, $P < 0.001$

Supplementary Table 1: Correlation analysis between AKT3 expression in GC and clinicopathological characteristics

Clinicopathological characteristics		Number of cases	Mean[1]	SD[2]	P-value
Gender	Male	22	-1.1674	4.2041	0.2197
	Female	7	-0.0987	2.5735	
Age	≤60	16	-0.8577	3.0912	0.8947
	>60	13	-0.9578	4.9667	
Vascular invasion	Positive	15	-0.9235	4.9176	0.9693
	Negative	14	-0.8944	3.1492	
Tumor location	Cardia	5	-0.8571	3.1749	0.9288
	Gastric body	3	-0.5000	3.1205	
	Gastric antrum	21	-0.9804	4.5115	
Borrmann type	I+II	15	-0.9097	4.5902	0.9995
	III+IV	14	-0.9092	3.5023	
pT stage	T1+T2	1	-5.7605	/	
	T3+T4	28	-0.7362	/	
pN stage	N0+N1	12	-1.5044	6.005	0.1785
	N2+N3	17	-0.4895	2.2806	
pM stage	M0	28	-0.9143	/	
	M1	1	-0.774	/	
pTNM stage	I+II	6	-2.7928	3.7865	0.064
	III+IV	23	-0.4182	2.9104	
Histologic grade	well	10	-1.2253	4.8870	0.542
	poor	19	-0.7432	3.5715	

[1] Mean of Log_2 (C/N); [2] Standard Definition of Log_2 (C/N); C: normalized expression of cancer tissues; N: normalized expression of adjacent noncancerous tissues.
